# Complex Oncological Decision-Making Utilizing Fast-and-Frugal Trees in a Community Setting—Role of Academic and Hybrid Modeling

**DOI:** 10.3390/jcm9061884

**Published:** 2020-06-16

**Authors:** Ravi Salgia, Isa Mambetsariev, Tingting Tan, Amanda Schwer, Daryl P. Pearlstein, Hazem Chehabi, Angel Baroz, Jeremy Fricke, Rebecca Pharaon, Hannah Romo, Thomas Waddington, Razmig Babikian, Linda Buck, Prakash Kulkarni, Mary Cianfrocca, Benjamin Djulbegovic, Sumanta K. Pal

**Affiliations:** 1 Department of Medical Oncology and Therapeutics Research, 1500 E Duarte Road, City of Hope National Medical Center, Duarte, CA 91010, USA; Imambetsariev@coh.org (I.M.); titan@coh.org (T.T.); abaroz@coh.org (A.B.); jfricke@coh.org (J.F.); rpharaon@coh.org (R.P.); hromo@coh.org (H.R.); rbabikian@coh.org (R.B.); lbuck@coh.org (L.B.); pkulkarni@coh.org (P.K.); mcianfrocca@coh.org (M.C.); spal@coh.org (S.K.P.); 2 Newport Diagnostic Center, Newport Beach, CA 92660, USA; aschwer@newportdiagnosticcenter.com (A.S.); h.chehabi@newportdiagnosticcenter.com (H.C.); 3 Department of Thoracic Surgery, Hoag Hospital, CA 92660, USA; darylpearlstein@gmail.com; 4 Department of Medicine, City of Hope National Medical Center, Duarte, CA 91010, USA; twaddington@coh.org; 5 Department of Hematology & Hematopoietic Cell Transplantation, City of Hope National Medical Center, Duarte, CA 91010, USA; bdjulbegovic@coh.org

**Keywords:** non-small cell lung cancer, actionable mutations, next-generation sequencing, fast-and-frugal trees, community practice, personalized medicine

## Abstract

Non-small cell lung cancer is a devastating disease and with the advent of targeted therapies and molecular testing, the decision-making process has become complex. While established guidelines and pathways offer some guidance, they are difficult to utilize in a busy community practice and are not always implemented in the community. The rationale of the study was to identify a cohort of patients with lung adenocarcinoma at a City of Hope community site (n = 11) and utilize their case studies to develop a decision-making framework utilizing fast-and-frugal tree (FFT) heuristics. Most patients had stage IV (N = 9, 81.8%) disease at the time of the first consultation. The most common symptoms at initial presentation were cough (N = 5, 45.5%), shortness of breath (N = 3, 27.2%), and weight loss (N = 3, 27.2%). The Eastern Cooperative Oncology Group (ECOG) performance status ranged from 0-1 in all patients in this study. Distribution of molecular drivers among the patients were as follows: EGFR (N = 5, 45.5%), KRAS (N = 2, 18.2%), ALK (N = 2, 18.2%), MET (N = 2, 18.2%), and RET (N = 1, 9.1%). Seven initial FFTs were developed for the various case scenarios, but ultimately the decisions were condensed into one FFT, a molecular stage IV FFT, that arrived at accurate decisions without sacrificing initial information. While these FFT decision trees may seem arbitrary to an experienced oncologist at an academic site, the simplicity of their utility is essential for community practice where patients often do not get molecular testing and are not assigned proper therapy.

## 1. Introduction

Lung cancer is a devastating disease with an overall survival rate of 16% at five years in non-small cell lung cancer (NSCLC) and 6% in small cell lung cancer (SCLC) [1]. Lung cancer incidence in 2020 is estimated at 228,820 new cases and will remain the leading cause of cancer death in the US with an estimated 135,720 deaths [2]. NSCLC accounts for approximately 85% of lung cancer cases and is a heterogeneous group of tumors comprised of adenocarcinomas, squamous cell carcinomas, and large cell carcinomas as the major subtypes [3]. These individual subtypes have different molecular characteristics, clinical features, and complex therapeutic options with unique outcomes for individual patients. More so, the advent of targeted therapy and immunotherapy have drastically shifted the therapeutic landscape of NSCLC towards precision medicine, or personalized medicine, where the individual patient is treated selectively based on their molecular characteristics [4]. Due to the rise of personalized medicine, lung cancer decision-making has grown complex, and chemotherapy, once the default treatment of choice for all patients, is no longer universally used.

This advance towards personalized medicine is primarily driven by advances in targeted therapeutic options, genomic testing, and immunotherapy that have shown improvement in patient outcomes [5,6,7,8,9,10]. EGFR therapy, once limited to first-generation tyrosine-kinase inhibitors (TKIs) such as erlotinib [11], has grown to second and third generation TKIs with osimertinib showing improvements not only in response rates and progression-free survival (PFS), but also in durable overall survival (OS) benefits as compared to chemotherapy and other TKIs [12,13,14]. These advances in therapeutic options for *EGFR* has further spurred greater understanding of NSCLC biology to reveal multiple distinct molecular subtypes—a widely supported theory that oncogenic “driver mutations” are responsible for NSCLC’s malignant phenotype [15]. Along with *EGFR,* gene alterations in *ALK*, *ROS1*, *NTRK*, and *BRAF* have therapies that are FDA-approved and routinely tested in practice [16]. Recent long-term follow-up results from 110 ALK-positive patients who received the ALK inhibitor crizotinib, showed a median OS of 6.8 years in patients with metastatic disease [17]. Moreover, several inhibitors for MET and RET are slowly being implemented in standard clinical practice and await FDA approval [18,19,20,21,22]. Initial results from a phase II study of MET exon 14 inhibitor tepotinib, showed a sustained duration of response (12.4 months) and a 45% objective response rate (ORR), earning a breakthrough FDA designation [23,24]. Beyond these driver alterations, approximately 25% of lung adenocarcinomas present with KRAS alterations that occur in codons 12 or 13 [25]. No approved direct targeted therapy is available for KRAS-mutant NSCLC, but two promising inhibitors—MRTX849 and AMG 510—are currently under clinical consideration with early results reporting antitumor activity in the initial patients [26,27]. Furthermore, several studies have shown that immunotherapy is a viable treatment option for KRAS-mutant patients, with some patients showing remarkable clinical benefit from anti-PD-1/PD-L1 immunotherapy [28,29,30].

The availability of several targeted therapies and a variety of immunotherapies that have been approved for lung cancer has complicated oncology decision-making in clinical practice. Furthermore, advances in genomics and therapies have not been accompanied with advances in the science of medical decision-making. Clinical practice guidelines, such as the National Cancer Center Network (NCCN) and the American Society for Clinical Oncology (ASCO), are commonly used to aid in decision-making, but the recommendations are often difficult to interpret and are not easily manageable during a busy oncology clinic [31,32]. We have previously shown that a novel method of utilizing fast-and-frugal trees (FFTs) to arrive at decision-making trees can allow for physicians to make a quick but accurate decision [33]. Our previous work shows how guidelines can be converted into FFTs to allow development of individualized patient care, and the quantitative analysis of the performance characteristics of such a strategy [34,35]. The advantage of FFTs is that they are highly effective, simple decision trees composed of sequentially ordered cues (tests) and binary (yes/no) decisions formulated via a series of “if-then” statements [34]. Therefore, they can be easily applied to lung cancer decision-making where, if a genetic alteration is revealed to be actionable (e.g., EGFR exon 19 deletion), then an appropriate TKI (e.g., osimertinib) would be given [33]. While these decisions may seem arbitrary to an experienced oncologist at an academic site, the simplicity of their utility is essential for community practice where patients often do not get molecular testing and are not assigned proper therapy.

Thus, to evaluate clinical decision-making in lung cancer in the community, we have selected a cohort of community practice patients and have described their clinical complexity in detail as well as summarized the process for clinical decision-making that was employed to arrive at best the outcome for the patients. The majority of patients in this study were advanced NSCLC patients and we wanted to explore the role of personalized medicine within the community setting. We have also utilized the FFT method to develop individual simplified decision-trees that can be utilized in similar oncology cases based on patient molecular and clinical characteristics.

## 2. Patients and Methods

### 2.1. Patients

The first eleven non-small cell lung cancer patients (n = 11), evaluated at City of Hope Newport Beach community practice since its opening in January 2020, were enrolled in this analysis. De-identified patient data was obtained under the City of Hope institutional review board-approved protocol IRB 18008 with a waiver of informed consent and in accord with the Declaration of Helsinki. Data obtained from the electronic medical record included patient demographics, stage, age at diagnosis, race and ethnicity, smoking history, date of diagnosis, therapies given, histology, and outcomes. Molecular testing results were obtained from a retrospective chart review based on next-generation sequencing (NGS) tests obtained by their primary oncologist.

### 2.2. Fast-and-Frugal Trees

Heuristic categorization and the visualization of fast-and-frugal trees was performed according to established heuristic tree construction algorithms as previously described [36,37].

## 3. Results

### 3.1. Patients

A total of 28 patients diagnosed with cancer of the lung or bronchus were seen from January 2020 through to April 2020 at COH Newport Beach community clinic; the first 11 patients were included (Table 1). The median age at diagnosis was 70.5 years, ranging from 50–85 years. More than half of the patients were male (N = 7, 63.6%), most were white (N = 6, 54.5%), and less than half had a history of smoking (N = 5, 45.5%), with an average of 10.2 pack years. Upon initial diagnosis, all 11 patients were histologically identified with adenocarcinoma. However, one patient had a small cell lung cancer transformation status after two lines of TKI therapy (not shown on table), but also retained the original lung adenocarcinoma histology. Most patients had stage IV (N = 9, 81.8%) disease at the time of the first consultation. The most common symptoms at initial presentation were cough (N = 5, 45.5%), shortness of breath (N = 3, 27.2%), and weight loss (N = 3, 27.2%). The Eastern Cooperative Oncology Group (ECOG) performance status ranged from 0–1 in all patients in this study. Distribution of molecular drivers among the patients were as follows: *EGFR* (N = 5, 45.5%), *KRAS* (N = 2, 18.2%), *ALK* (N = 2, 18.2%), *MET* (N = 2, 18.2%), and *RET* (N = 1, 9.1%). Patients who tested positive for PD-L1 expression (N = 5, 45.5%) were considered positive if their tumor proportion score was greater than or equal to 1%.

### 3.2. Genomics

**Figure 1:**
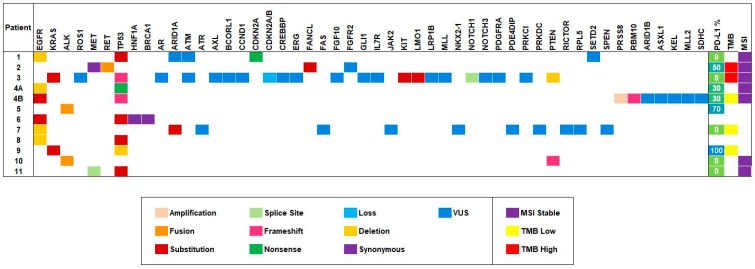
Mutational profile of the 11 patients as represented by heatmap. The figure displays tumor alterations reported by next-generation sequencing (NGS) testing, with the most frequent alterations on the left of the heatmap. The most frequent alteration types include substitution, deletions, and VUS mutations. The heatmap on the right reports the PD-L1, TMB, and MSI status of the cohort. Patient 4 is noted twice in the heatmap due to NGS testing performed on synchronous primary lung tumors (4A is the right lung specimen mutational profile; 4B is the left lung specimen mutational profile).

There were ten types of mutations identified in this cohort of patients. Eleven (100%) patients underwent NGS testing performed on tumor and/or liquid biopsies. Most patients had more than one genomic alteration reported. Figure 1 presents a heatmap of the biomarkers reported per patient, including PD-L1, TMB, and MSI status. Overall, the most frequent mutations found in this cohort were *TP53* (8/11, 66.7%), *EGFR* (5/11, 45.5%), *ALK* (2/11, 18.2%), *KRAS* (2/11, 18.2%), and *MET* (2/11, 18.2%). One patient, patient #4, is included twice in the heatmap due to NGS testing performed on two simultaneous, biologically different lung tumors (4A NGS was performed on the right lung specimen; 4B NGS was performed on the left lung specimen). The most frequent mutation types found in this cohort were substitution mutations (noted in red), deletions (noted in orange), and variant of unknown significance (VUS) mutations (noted in blue).

### 3.3. EGFR

#### 3.3.1. Case #1

A 63-year-old Caucasian male never-smoker with a history of coughing that initially presented as throat clearing and progressed to regular frequent cough. He then developed a mild left-sided chest wall pain that improved over time, but the patient reported weight loss of about 30 pounds. He underwent a bronchoscopy that noted marked narrowing of the right middle lobe (RML) by extrinsic compression. A biopsy revealed lung adenocarcinoma of the RML, and a PET scan revealed a hypermetabolic 2.5 × 2.2 cm lesion in the left upper lobe (LUL). Molecular testing was performed on samples from both sides and showed differing results. The left-sided biopsy molecular testing showed EGFR L858R, PRSS8 amplification, RBM10 I377fs*4, and TP53 S90fs*33. The right-sided lesion showed EGFR amplification, EGFR exon 19 deletion (E746_A750del), and TP53 R306* alteration. The patient was discussed at the tumor board, and as surgery was not an option, the decision was made to treat the patient as stage IV EGFR lung cancer with a bilateral disease, and erlotinib therapy was immediately started. He was staged as a T1cN2M1a stage IVA lung adenocarcinoma. The patient had a good initial response on the right side and maintained stable disease on the left side for over 13 months. He eventually developed excess pleural effusion and liquid biopsy molecular testing revealed an EGFR T790M resistance alteration. A PleurX catheter was installed and the patient was immediately switched to osimertinib. He tolerated osimertinib well and continued on therapy for approximately 14 months until a PET/CT scan showed a mild interval increase in size of the hypermetabolic right cardiophrenic lymph node. This was considered oligoprogression, as the overall disease burden was stable and osimertinib therapy continued. A right supraclavicular, right mammary node, and a right cardiophrenic mass resection was performed and pathology showed small cell lung cancer, synaptophysin positive. Molecular testing was performed, and results showed EGFR exon 19 deletion (E746_A750del), EGFR amplification, AKT1 amplification, and TP53 R306*. EGFR TKI-small cell lung cancer transformation was suspected, and the patient immediately started carboplatin/etoposide chemotherapy alongside osimertinib. The patient tolerated combination therapy well and continues on therapy.

#### 3.3.2. Case #2

A 66-year-old Asian female never-smoker initially presented with left shoulder pain and numbness. She underwent a chest X-ray which showed an abnormality in the lingula of the left-upper lobe. A CT-guided biopsy was performed and revealed TTF-1 positivity, moderately differentiated pulmonary adenocarcinoma with a predominant acinar pattern. Staging CT of the chest, abdomen, and pelvis displayed a 1.5 × 2.0 × 2.2 cm left upper lobe mass. A subsequent PET/CT demonstrated F-18 fluorodeoxyglucose (FDG) activity in the left upper lobe, but also in multiple mediastinal and left hilar lymph nodes including stations 4R, 4L, 7, and 11R. These would be later confirmed for metastatic adenocarcinoma after she underwent an endobronchial ultrasound with fine needle biopsy. NGS sequencing of the station 7 lymph node revealed EGFR exon 19 deletion (E746_A750del), CDKN2A G120*, and TP53 Y163C. PD-L1 22C3 stained negative with 0% expression. The PET/CT also exhibited a 1.1 cm calcified isthmus thyroid gland nodule with an SUV of 4.9. This was also biopsied and revealed papillary carcinoma. She was referred to the endocrine surgery department but has not had surgery considering her lung cancer diagnosis. An MRI of the brain showed no metastatic disease. She was staged as a T1cN3M0 stage IIIB lung adenocarcinoma. She initiated therapy on weekly chemoradiation therapy to the left lung, hilum, and mediastinum with carboplatin and paclitaxel. She completed radiation with a few acute toxicities including chest discomfort, fatigue, and esophagitis, for which she received a 10-day course of dexamethasone. Due to her acute toxicities, she only received three weekly infusions of carboplatin and paclitaxel. She is planned to continue systemic treatment by initiating adjuvant osimertinib, and while this is not yet approved, the decision was made based on preliminary trial data and the factor that the patient could not complete chemotherapy due to toxicities.

#### 3.3.3. Case #3

An 86-year-old male with no history of tobacco use initially presented to the emergency department with a complaint of dry cough, shortness of breath, and concern of gradual weight loss. He developed a dry cough about two months prior, without any specific or potential triggering cause. The persistent shortness of breath occurred over a few days, however, without limitations to his usual routines. Chest X-ray showed extensive consolidation and effusion on the right. Subsequent CT chest with IV contrast revealed severe right-sided pleural effusion, multiple nodular and patchy opacities in the right lung, with possible combination of atelectasis and edema. There were multiple sub-centimeter lesions throughout the lungs bilaterally, suspicious for pulmonary metastasis of more than 20 lesions. During his admission, he underwent thoracenteses that identified rare atypical cells suggestive of adenocarcinoma with lung primary (TTF-1 positive, Napsin A positive, mucicarmine cytochemistry negative). His disease progressed one month after admission. PET/CT confirmed diffuse FDG avid right pulmonary consolidation and bilateral pulmonary nodules with a max SUV of 11.6, FDG avid cervical and thoracic lymphadenopathy, and FDG avid osseous lesions. Brain MRI showed a motion-limited exam without findings to suggest intracranial metastatic disease. Single gene NGS panels showed EGFR exon 21 L858R mutation. Approximately 2.5 months after diagnosis, the patient had an initial consultation at COH community site. He was staged as a T2N3M1c stage IVB lung adenocarcinoma. He was treatment-naïve prior to consultation. Osimertinib was immediately administered after liquid biopsy confirmed EGFR exon 21 L858R and TP53 R273L. However, he experienced severe diarrhea 19 days after treatment start date. Osimertinib was held for 20 days and dosing was reduced to 40 mg daily. A few weeks following treatment re-initiation, the patient was admitted to the hospital for worsening shortness of breath associated with decreased output from his pleural catheter. Oxygen saturation ranged from 88–92% on room air. He transitioned from supplemental oxygen to high flow oxygen-enriched FiO2. Empiric antibiotics were administered for hospital-acquired pneumonia. His respiratory distress continued to deteriorate with drops in oxygen saturation, requiring continuous BIPAP treatment and nebulizer therapy. When he required BIPAP support, his family decided on comfort care. In combination with his comorbidities of atrial fibrillation, chronic respiratory failure with hypoxia, and bilateral DVT of lower extremities, the patient unfortunately passed away 23 weeks after diagnosis due to post-obstructive pneumonia and respiratory complications.

#### 3.3.4. Case #4

A 63-year-old male never-smoker with a history of well-controlled type II diabetes without retinopathy initially presented to his ophthalmologist with blurred vision over the left eye with intermittent, mild irritation ongoing for one month. He denied any focal neurological deficits. On examination, an amelanotic choroidal lesion with subretinal fluid on the left eye was prominent and appeared malignant. CT of chest revealed a large spiculated centrally necrotic right upper lobe mass that extended to the suprahilar region measuring approximately 5.0 × 4.3 cm. There were enlarged centrally necrotic conglomerated right lower paratracheal lymph nodes, measuring up to 20 mm in maximum short axis. Subsequent CT of chest, abdomen, and pelvis demonstrated an indeterminate 1.6 cm left adrenal nodule, and an indeterminate 8 mm lucent lesion in the L4 spinous process, metastasis not excluded. The patient followed up with pulmonologist and his symptoms of mild fatigue and recent weight loss were noted. Further workup involved endobronchial ultrasound bronchoscopy of right upper lobe mass and lymph node stations. Pathology showed adenocarcinoma immunohistochemically consistent with lung primary (TTF-1 positive, Napsin A positive, CK7 positive, CK20 negative, synaptophysin negative, chromogranin negative). PET/CT confirmed the large FDG avid right upper lobe pulmonary mass extending to the right suprahilar region measuring 7.7 SUV, ground glass and consolidative opacities involving right upper lobe, FDG right level 4 and mediastinal lymph nodes, FDG avid osseous lesions involving the T1 vertebral body, right posterior iliac bone and left acetabulum, and a 14 mm FDG avid left adrenal nodule. Brain MRI showed 4 mm enhancement in the left superior frontal gyrus, concerning for metastasis. He had his first initial consultation at COH community site approximately two months after his ophthalmology visit. He was staged as a T3N2M1c stage IVB lung adenocarcinoma. Complete NGS testing demonstrated EGFR exon 19 deletion (L747_A750 delinsP). No other driver mutations were found. Osimertinib was administered six weeks after pathologic diagnosis. Patient continues on medication and osimertinib is well tolerated. A recommendation to treat brain metastasis with radiation was withdrawn due to osimertinib’s CNS penetration.

#### 3.3.5. Case #5

**Figure 2:**
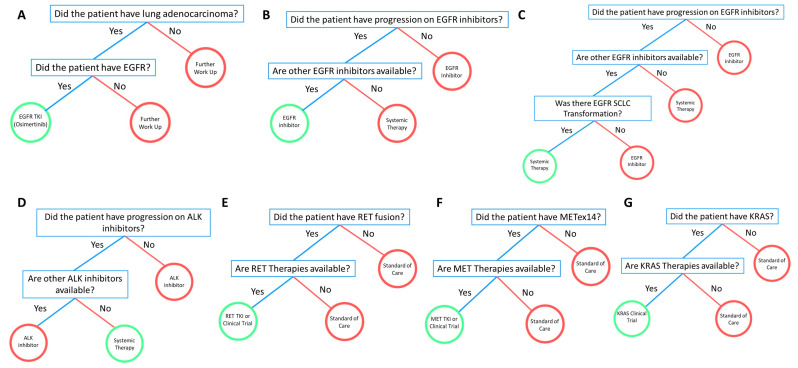
Fast-and-Frugal trees developed from community practice cases. (**A**–**C**) Representative fast-and-frugal trees for EGFR-mutated patient case scenarios. (**D**) Fast-and-frugal tree applicable to ALK mutated patient scenarios. (**E**) Fast-and-frugal tree for RET decision-making. (**F**) Fast-and-frugal tree for MET decision-making. (**G**) KRAS mutated fast-and-frugal decision-making tree.

A 50-year-old Asian male never-smoker presented with a productive cough and clear sputum with specks of blood approximately two years ago. His primary care doctor directed him to his gastroenterologist with the belief that the cough was secondary to gastroesophageal reflux disease. A chest X-ray ordered by the gastroenterologist revealed right lower lobe consolidation. The patient underwent a CT scan of the chest, abdomen, and pelvis a month later which showed a moderate mass-like consolidation and multifocal nodular opacities in the right lower lobe, possibly obscuring an underlying mass. Additionally, there were bilateral small 0.4 cm lung nodules, a 0.7 cm AP window lymph node, a 1.3 cm subcarinal lymph node, and a 2.2 cm right infra-hilar lymph node. The patient underwent further workup in hospital. A sputum test ruled out tuberculosis and a CT-guided lung biopsy was negative for malignancy. The patient then underwent a transbronchial lung bronchoscopy which also did not reveal malignancy. However, the bronchial brushing and washing demonstrated malignant cells consistent with adenocarcinoma with weak TTF-1 positive staining. Two months later, the patient endorsed chest wall pain and shortness of breath after long periods of coughing. An MRI scan of the brain demonstrated no evidence of brain metastasis. A PET/CT scan performed revealed strong FDG uptake in mediastinal lymph nodes, small right supraclavicular lymph nodes, and hypermetabolic pulmonary nodules bilaterally. He was staged as a TXN3M1a stage IVA lung adenocarcinoma. Molecular testing was performed and showed EGFR exon 19 deletion (E746_A750del) and TP53 286K mutation. Osimertinib was administered with good tolerance. Figure 2A–C shows the representative FFTs developed from the EGFR cases above.

### 3.4. ALK

#### 3.4.1. Case #1

A 67-year-old Caucasian female never-smoker presented to City of Hope with an extensive history of lung cancer treated with several lines of therapy. She was staged as a T2N3M1c stage IVB lung adenocarcinoma. Initially, the patient was treated with carboplatin, taxol, and bevacizumab, but did not tolerate it well and continued pemetrexed maintenance. A few years after diagnosis, she was found to have progression and metastatic disease in the right ribs and chest wall. Molecular testing revealed an ALK-positive tumor. The patient was then started on crizotinib, ceritinib, and alectinib in sequence. Ceritinib was poorly tolerated due to significant gastrointestinal symptoms and she was switched to alectinib which she continued on for two years until the disease progressed. The patient was then started on lorlatinib and received three courses of radiation therapy due to the growing chest wall lesion. The chest wall mass measured as 2.5 to 3 cm and the patient reported pain due to the lesion. Pemetrexed was added to lorlatinib during this time. She subsequently presented to City of Hope with increased pain in the chest wall lesion with it visibly growing on the chest. Pemetrexed and lorlatinib were placed on hold as the patient had become anemic and neutropenic secondary to therapy. Due to the growing chest wall lesion, she was switched to docetaxel and ramucirumab, while also receiving 25 Gy in five fractions to the chest wall mass. The patient unfortunately developed a growing pelvic mass on the right pelvis and therapy was switched to ceritinib, as her records reported a good response to ceritinib in the past, although she did suffer from toxicity. There was improvement in the size of the chest wall lesion and the patient continues ceritinib therapy almost ten years after her initial diagnosis.

#### 3.4.2. Case #2

An 81-year-old Asian male former smoker (12.5 pack years) presented with a history of pT3N0 poorly differentiated gastric adenocarcinoma. A robotic-converted-to-open-gastrectomy with D1+ lymphadenectomy was performed. Following the surgery, he declined adjuvant therapy and was followed on active surveillance for almost two years. During surveillance imaging, he was found to have an enlarged lower lung nodule, although he was asymptomatic for lung cancer at that time. An MRI scan of the brain demonstrated no evidence of metastatic disease within the brain. A staging PET/CT scan showed an avid mass in the posterior medial left lower lobe lung measuring up to 31 × 21 mm. The patient underwent a flexible bronchoscopy with robotic assisted left lower lobectomy and mediastinal lymph node dissection. The results revealed adenocarcinoma of lower lobe of the left lung measuring approximately 3.5 × 3 × 2.5 cm in size, with one lymph node involved. The initial immunohistochemistry performed on the specimen was positive for CK7, TTF-1, and Napsin A. Following surgical resection, the patient endorsed symptoms of productive cough with clear sputum, fatigue, poor appetite, and weight loss. Molecular testing was performed and revealed EML4-ALK fusion, PTEN R74Tfs, and SMARCA4 D696Vfs*3. He was staged as a T2aN2M0 stage IIIA lung adenocarcinoma. The patient was planned to initiate alectinib, but has not yet begun due to fear of side effects (Figure 2D). While adjuvant alectinib is not yet officially approved, the patient was deemed a poor candidate for chemotherapy due to his frailty and did not wish to have chemotherapy. Thus, based on the ALK mutation, alectinib was recommended due to the early trial results.

### 3.5. RET

#### Case #1

An 80-year-old Caucasian male with a light smoking history (<1 pack years) developed a cough, and a chest X-ray was performed that showed a persistent right upper lobe (RUL) airspace opacity consistent with pneumonia. A week later, a chest CT showed a 2.5 × 3.2 × 5 cm right upper lobe consolidation extending from the right suprahilar region to the right lung apex, and a narrowing of the right mainstem bronchus. There were also multiple right and left sub-centimeter nodules, and lesions within the superior aspect of the T4 and T9 spine. He underwent a CT-guided biopsy of the RUL which was consistent with moderate to poorly differentiated lung adenocarcinoma staining positive for TTF-1 and Napsin A and negative for p63. Partial molecular testing was negative for EGFR mutations or ALK rearrangements but stained positive for PD-L1 at 50%. Further work-up with a diagnostic PET/CT also showed an FDG-avid hepatic lesion. A brain MRI was unremarkable, with no evidence of metastatic disease. A CT-guided biopsy of the T9 lesion confirmed metastatic lung adenocarcinoma and was used for complete molecular profiling. This test revealed a RET-KIF5B fusion, TMB-high with 11 Muts/Mb, and FANCL exon 12 c.1020 + 2T > C detected. He was staged as a T3N2M1c stage IVB lung adenocarcinoma. The patient started first line systemic therapy with a combination of carboplatin, pemetrexed, and pembrolizumab and received four cycles before starting maintenance pemetrexed/pembrolizumab. He received nine maintenance cycles before developing diarrhea. A CT of the chest, abdomen, and pelvis showed rectosigmoid and proximal transverse colitis for which he was treated with prednisone, and maintenance therapy was discontinued. He remained off therapy for six months before a CT of the chest, abdomen, and pelvis showed new low-density hepatic lesions and pathologic compression fractures at T4 and T9, consistent with progressive disease. He was screened and consented for a clinical trial for the expanded access of selpercatinib (LOXO-292) for RET fusions; however, he needed to control his hypertension prior to trial initiation. After multiple adjustments of his hypertensive medications by his cardiologist, he was able to start treatment with selpercatinib (Figure 2E).

### 3.6. MET

#### Case #1

A 70-year-old female never-smoker with a past medical history of arthritis presented to an outside institution with shortness of breath and a cough. CT abdomen was performed and showed a small right pleural effusion and no evidence of an acute intra-abdominopelvic process. The patient underwent a PET/CT scan which demonstrated a medial right apical hypermetabolic pulmonary mass measuring approximately 3.1 x 2.7 cm (SUV max 12.5), with extension into the adjacent pleura, multiple additional hypermetabolic pleural-based soft tissue masses within the right hemithorax, interval mild to moderate increase in right-sided pleural effusion, and no evidence of distant metastatic disease. A CT-guided biopsy was performed on the right upper lobe mass and revealed lung adenocarcinoma. Both NGS tests demonstrated MET exon 14 splice site mutation (2888-42_2892del47) (MAF: 1.1%), TP53 G334V missense mutation (MAF: 1.5%), MSI Stable, TMB-low (9 Muts/Mb), and PD-L1 0%. She underwent an MRI of her lumbar and thoracic spine that showed a loculated pleural effusion with enhancing pleural lesions compatible with metastatic disease, lytic changes to the medial right 12th rib compatible with osseous metastatic disease, disk bulging was incidentally noted at T8, and a nonspecific 0.6 cm lesion at L1. Brain MRI at the time was negative for metastatic disease. She was staged as a T3N2M1c stage IVB lung adenocarcinoma. The patient was subsequently enrolled in a clinical trial investigating MET inhibitor capmatinib 400 BID and continued with symptomatic improvement with the first restaging scan showing response. The second interval scan showed progression with a slight increase in the right apical pleural-based mass, multiple right basilar pleural-based lesions, and a loculated right pleural effusion. The patient underwent liquid NGS testing that revealed MET exon 14 splice site (2888-42_2892del47) (MAF: 0.63% deceased from 1.1%) and TP53 G334V (MAF: 0.78% decreased from 1.5%). Progression of disease was confirmed on the third interval scan and the patient was taken off trial. She was subsequently started on carboplatin and nab-paclitaxel. Unfortunately, a restaging scan after two cycles showed interval progression of disease in the pleural-based mass, lymph nodes, and pleura. The patient presented to City of Hope for treatment options and was started on a clinical trial examining a bispecific monoclonal antibody targeting MET. She is status/post three cycles of the drug and is exhibiting stable disease thus far (Figure 2F).

### 3.7. KRAS

#### 3.7.1. Case #1

A 68-year-old Caucasian female former smoker (20 pack years) presented with a cough. An initial chest CT showed a 5.4 × 5.7 cm left upper lobe lesion with numerous bilateral sub-centimeter nodules. CT-guided core needle biopsy of the left upper lobe mass revealed moderately differentiated lung adenocarcinoma. Molecular profiling of the biopsy was negative for EGFR mutations, ALK rearrangements, ROS1 rearrangements, and PD-L1. Subsequent NGS of the biopsy revealed KRAS G12A, CDKN2A/B loss, LMO1 R34H, NOTCH1 splice site 2467 + 1G > TA, and TMB-intermediate with 13 Muts/Mb. A brain MRI showed a 2 mm lesion in the right prefrontal gyrus adjacent to the central sulcus of unclear etiology. In repeat brain MRIs, this lesion had resolved and was no longer present. To finish staging, a diagnostic PET/CT showed a 6 cm mass FDG avid with an SUV of 9.6 and multiple bilateral FDG avid pulmonary nodules. She was staged as a T3NXM1a stage IVA lung adenocarcinoma. She initiated first-line systemic therapy with cisplatin and pemetrexed for three cycles with very little response. Concurrent radiation to the left upper lobe primary and mediastinum was added to regimen with no significant toxicities. With no other signs of metastatic disease, she underwent a wedge resection and one of the pulmonary nodules showed metastatic disease. Thus, the rest of the procedure was aborted. She received second-line atezolizumab without disease progression for 17 months. A new CT-guided biopsy of the right lower lobe lung was performed and was confirmed as metastatic lung adenocarcinoma. NGS molecular profiling revealed KRAS G12A, KRAS L19F, KIT A829S, PDGFRA T365N, PTEN deletion, and TMB-high with 11.4 Muts/Mb. She started third-line systemic therapy with carboplatin, paclitaxel, bevacizumab, and pembrolizumab for a total of five cycles. She is currently status/post seven cycles of maintenance bevacizumab and pembrolizumab without signs of disease progression.

#### 3.7.2. Case #2

An 80-year-old Caucasian male never-smoker had a history of hairy cell leukemia that was treated approximately 20 years ago with chemotherapy (cladribine) for eight days and continues to follow up regularly. The patient was diagnosed with early stage lung cancer two years ago, following a biopsy of a lung nodule in the right lower lobe. He had a platinum coil as fiducial marker placed at that time. A year later, the patient underwent radiation therapy for a malignant neoplasm of the lower lobe right bronchus. Unfortunately, his disease progressed, and the patient was deemed non-operable due to medical comorbidities including diabetes mellitus, a history of coronary artery disease, and a history of hairy cell leukemia. A PET/CT scan performed ten months after radiation revealed a hypermetabolic right lower lobe pulmonary mass with central metallic fiducial marker unchanged in size and metabolic activity compared to a prior study with increased right pleural effusion. A thoracentesis performed shortly after demonstrated malignant cells seen as single cells, comprising 1–2% cellularity. Following the thoracentesis procedure, the patient reported improvement to his shortness of breath. Additionally, he reported neuropathy secondary to diabetes mellitus onset eight years ago, which is controlled, and he continues to follow up with endocrinology. He was staged as a T2NXM1a stage IVA lung adenocarcinoma. Molecular testing was performed, and results revealed BRAF V600E, NRAS G12D, KRAS G12S, PDL1 100%, meanwhile the BRAF V600E IHC was negative. The patient was subsequently initiated on pembrolizumab. One week after his first cycle, he was admitted to hospital for pneumonitis, chest pain, onset fever, transaminitis, and elevated blood pressure without previous diagnosis of HTN, thrombocytopenia, and nephrolithiasis. He was treated with IV Rocephin, oral oxycline, pain control, and given a pulmonary consultation. The patient was discharged with antibiotics. The patient continues on pembrolizumab with continued symptoms of fatigue and decreased appetite (Figure 2G).

## 4. Discussion

Between 2013–2017, there were 68,369 total cases of invasive cancers and 6,541 cases of lung cancer in Orange County, according to the California Cancer Registry. Age-adjusted invasive cancer incidence demonstrates that Orange County accounted for higher cancer rates compared to Los Angeles County (394.93 vs. 371.36 per 100,000 cases, respectively). Similarly, age-adjusted lung cancer incidence rate in Orange County is higher than in Los Angeles county (38.29 vs. 35.88 per 100,000 cases, respectively) [38]. Located in Orange County, Newport Beach is the newest community site in the City of Hope network. Demographic information was assessed for new patients in the first four months of operation, beginning in January 2020. Only initial new patient visits were accounted for, thereby excluding subsequent follow-up visits from the count. We identified 182 new patients, 118 (64.8%) of whom were female and 64 (35.2%) of whom were male. The median age of the patients was 63 years, ranging from 22 to 90 years. The most prevalent ethnicities were Caucasian (n = 96, 52.7%) and Asian (n = 16, 8.8%). Among the 182 patients, 28 were diagnosed with cancer in the lung or bronchus, 11 of whom were presented in this report.

Fast-and-frugal trees (FFTs) are an effective way to make binary classification decisions under time pressure conditions with only limited information [36,39]. One example to illustrate this is the decision of whether to send a patient to coronary care or a regular hospital bed if a patient reports chest pain [40]. FFTs can be used to evaluate standard guidelines and establish new decision-making heuristic strategies, allowing physicians to make fast and accurate decisions without relying on statistical training or calculation devices. An FFT is a decision tree composed of sequentially ordered cues such as diagnostic test results or clinical information and the accompanying binary decision (yes/no) of those cues [36,41,42,43]. The relationship between the cues can be framed as “if-then” statements; for instance, if a patient has severe chest pain, then the physician should perform tests for myocardial infraction. If the condition of the cues is met, then the decision can be made, and the FFT exited. If the condition is not met, the FFT sequentially considers other cues, until a cue’s exit condition is met. The last cue of an FFT has two exits to ensure that a decision is ultimately made [36,41,42,43,44]. Formally, an FFT is defined as a decision tree that has m + 1 exits, with one exit for each of the first m-1 cues, and two exits for the last cue [36,41,42,43,44]. The FFT tool is robust, and it can be used to both evaluate established clinical pathways and guidelines, but also to develop new FFT-based decision-making trees that incorporate additional clinical data like molecular testing results. The identification of driver mutations for NSCLC has transformed patient care, such that patients who are properly screened with molecular testing have better outcomes than those who are not [45]. We wanted to develop an omics-based FFT that would outline the clinical course of action a physician should undertake when facing molecular cues.

**Figure 3:**
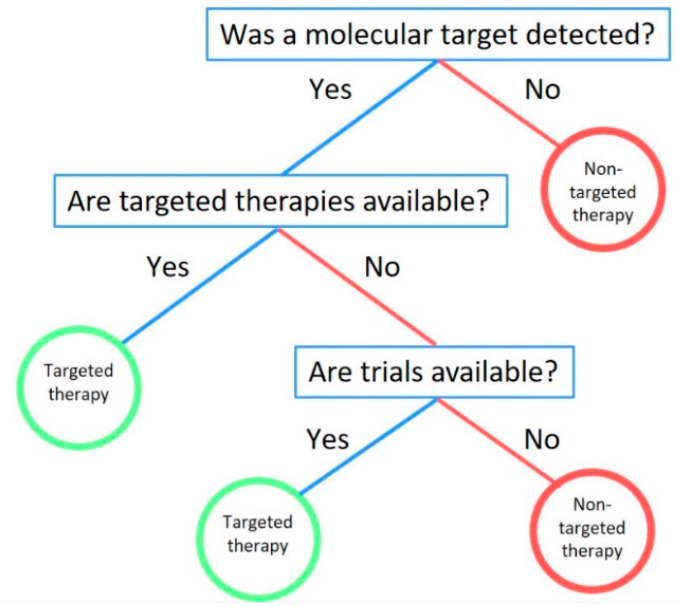
Comprehensive fast-and-frugal decision tree for lung cancer. A comprehensive molecular fast-and-frugal tree for stage IV lung cancer decision-making.

In our analysis, we had identified individual mutational subgroups to develop fast-and-frugal trees. From the five cases of *EGFR* patients, three FFTs were developed according to their individual cases. The heuristic steps in these FFTs were similar for most patients except for one who had small cell lung cancer transformation, which has been previously associated with prolonged EGFR TKI treatment [46,47]. However, the FFT was flexible enough and the addition of one additional step resolved the issue of systemic therapy (platinum/etoposide) for the SCLC transformation. These experiences were similar for the other molecular cases including *ALK*, *RET*, *MET*, and *KRAS*. Decisions could be made with either two or three queues without sacrificing the accurate decision based on limited information. As FFTs can be used to give form and structure to patterns, our analysis introduced a pattern regarding molecular decision-making. In each case, the final decision was dependent on the first question to be asked: “Was a molecular target detected?”. Without first answering that question, the oncologist cannot move on in their decision-making and should therefore consider ordering molecular testing. The subsequent question was also directly related: “Are targeted therapies available?”. This question would allow physicians to consider targeted therapies for all molecular markers in our analysis. Furthermore, we found this question to be important as it applied to even novel genetic targets such as KRAS whose targeted therapies are still under clinical trials. Therefore, the final question to be asked is: “Are trials available?”. The versatility of this question is that it allows an interpretation of genomic data not only based on FDA-approved therapies, but also on novel clinical trial drugs, such as KRAS inhibitor MRTX849 [26], that are still under evaluation. With these questions, we developed a comprehensive molecular decision-making FFT for stage IV lung cancer patients (Figure 3).

The advantage of a molecular FFT is that it applies to all molecular targets and while NCCN guidelines and other pathways have incorporated a few molecular targets such as EGFR, ALK, ROS1, and BRAF, they do not include MET, RET, NTRK or KRAS, for which therapies are quickly emerging and showing immense benefits to patients [48,49,50,51,52]. Our FFT would benefit community practice as it would incorporate those molecular targets into the decision-making strategy regardless of whether the therapy is FDA-approved or if it is in a clinical trial. The versatility of the FFT strategy shows that simple heuristics can be utilized efficiently to arrive at accurate decisions, and incorporating such FFTs into community practice can not only help with decision-making, but can also transform the decisions that oncologists make daily into a cognitive science.

### 4.1. EGFR

EGFR is a transmembrane protein that exists as a monomer on the surface of a cell. EGFR is a receptor tyrosine kinase, therefore it dimerizes with its specific ligand to activate the tyrosine kinase (TK) [53]. Once activated, the EGFR TK activates pathways which synthesize DNA and cause cell proliferation [54]. Thus, mutations or amplification of EGFR result in tumor growth/cancer. Predominant mutation locations include exon 19 deletions and exon 21 L858R point mutations [55]. EGFR mutations also occur more commonly in nonsmokers, women, and in people of Asian descent. A study demonstrated that EGFR mutations occurred in 42% of females compared to 14% of males and in 51% of nonsmokers compared to 10% of smokers [56]. EGFR TKIs have been heavily explored and single agent TKIs such as erlotinib, gefitinib (first generation), and afatinib (second generation) have become standard of care for EGFR mutant NSCLC patients. Osimertinib (a third generation TKI) emerged as a first-line treatment when it demonstrated improved outcomes when compared to outcomes with erlotinib and gefitinib in the FLAURA trial [57]. The FLAURA trial was conducted with 556 NSCLC patients with EGFR exon deletion or exon 21 L858R point mutation. Osimertinib showed improved PFS (18.9 vs. 10.2 months) and OS (83% vs. 71% at 18 months) when compared to standard of care treatment with gefitinib or erlotinib [58]. Dacomitinib, a second-generation single agent EGFR TKI, was approved by the FDA in 2018 as a first-line treatment for metastatic NSCLC based on the ARCHER 1050 trial [59]. The ARCHER 1050 trial compared dacomitinib to gefitinib in 452 patients with metastatic NSCLC and demonstrated a survival benefit in PFS (14.7 vs. 9.2 months) and OS (34.1 vs. 26.8 months) [60]. Several studies have demonstrated that patients who initially respond well to an EGFR TKI develop resistance, and subsequently their disease progresses [61,62,63]. Secondary EGFR mutations and MET amplifications are the most well-known causes of resistance [64,65]. Osimertinib selectively inhibits both EGFR-TKI-sensitizing and EGFR T790M resistance mutations, making it a valuable treatment against TKI-acquired resistance [12]. Alternative treatment options currently explored are combination therapies such as EGFR inhibitors along with bevacizumab or ramucirumab, vascular endothelial growth factor receptor inhibitors, EGFR inhibitors with chemotherapy, and EGFR inhibitors with other targeted therapy [66,67,68].

### 4.2. ALK

Initially identified in 2007, the formation of echinoderm microtubule-associated protein like-4 (EML4)-ALK fusion protein in NSCLC leads to constitutive oncogenic signaling. Patients with NSCLC harboring ALK translocations were estimated to be in approximately 6% of all NSCLC cases. Four years later, crizotinib became the first oral, ALK TKI approved by the FDA [69]. As a first-line treatment, crizotinib was shown to be safe and effective in a single arm, open-label, phase III clinical trial [70,71]. Among the patients who received prior systemic therapy, crizotinib showed an ORR of 50% and an improved quality of life compared to those on chemotherapy. PFS was improved for patients on crizotinib compared to standard of care chemotherapy by 3.9 months (10.9 vs. 7.0 months, respectively). However, the study showed that there was no difference in OS (approximately 12 months). Subsequently, other targeted therapies were developed as resistance eventually developed in advanced ALK-positive NSCLC patients. Acquired resistance mechanisms such as the amino acid substitution L1196M in the ALK gene against crizotinib have been explored [72]. Acting as a gatekeeper mutation, ALK L1196M impedes crizotinib’s efficacy in ALK-positive NSCLC patients. A hypothesis of resistance involves decreased binding energy within an unstable secondary structure of the mutant ALK complex [73]. The equivalence of this mutation is the EGFR mutation T790M that confers resistance to erlotinib or gefitinib [74]. Development of second-generation ALK inhibitors including ceritinib, alectinib, and brigatinib, were sequentially approved after crizotinib in 2011. Barrows et al. outlined observational studies of sequential treatment with several ALK inhibitors [75]. Effects from consecutive treatment with ALK inhibitors may be additive in terms of survival outcomes in patients. In order to overcome ALK resistance, the discovery of new ALK inhibitors is pivotal and essential in the clinical setting.

### 4.3. RET

Activation of the RET gene leads to cell proliferation, migration, and differentiation, and can be found on chromosome 10q11.2 [76,77]. The proto-oncogene *RET* interacts with multiple downstream pathways including JAK/STAT, MAPK/ERK, and PI3K/AKT [78]. In 1985, the RET rearrangement was seen for the first time when lymphoma DNA was transfected in NIH-3T3 cells [79]. Mutations and rearrangements in RET are most commonly observed in papillary thyroid carcinomas and in lung cancer [80]. RET fusions lead to tumorigenesis by continuous activation via ligand-independent dimerization and autophosphorylation of the RET kinase and its downstream partners [81]. RET most frequently fuses with KIF5B in NSCLC but other fusion gene partners have been observed in CCDC6, NCOA4, and TRIM33 [82]. In NSCLC, RET rearrangement is detected in 1–2% of cases, typically adenocarcinoma histology, and without a history of smoking [83,84]. Several multi-kinase TKIs have been utilized in targeting RET rearrangements in NSCLC. In a phase II study, cabozantinib, targeting VEGFR, MET, and RET, was shown to have an ORR of 28%, a median PFS of 5.5 months, and a median OS of 9.9 months in 26 RET fusion-positive NSCLC patients [20]. A phase II study of 17 RET fusion-positive NSCLC patients previously treated with chemotherapy received vandetanib, targeting VEGFR 2 and 3, EGFR, and RET, and the trial demonstrated a 53% ORR, a median PFS of 4.7 months, and a median OS of 11.1 months [85]. A new generation of novel RET inhibitors are currently being investigated such as selpercatinib (LOXO-292) and pralsetinib (BLU-667). The phase I/II LIBRETTO-001 trial investigating selpercatinib RET-rearranged NSCLC patients with previous therapy found an ORR of 68% [86,87]. Selpercatinib was recently awarded FDA approval for adult patients with RET-positive NSCLC based on the previous trial with an ORR of 85% in treatment of naïve patients [88]. In a phase I study of pralsetinib, an ORR of 58% was demonstrated for all patients regardless of RET fusion type and was effective against intracranial metastases [89].

### 4.4. MET

*MET* proto-oncogene encodes for c-Met, a member of the receptor tyrosine kinase (RTK) family. Once activated, c-MET is involved in various signaling cascades linked to cell growth, apoptosis, motility, and invasion. Mutations in MET represent 6% of all NSCLC cases with frequent alterations including MET exon 14 skipping mutation and MET amplification [90]. Since its discovery in lung cancer and other solid tumor malignancies, research has been investigating potential targeting of MET through small molecule inhibitors or monoclonal antibodies. Initially, a phase II clinical trial enrolled recurrent NSCLC, which were randomized to receive onartuzumab with erlotinib or placebo with erlotinib [91]. In NSCLC patients with MET-mutated disease, treatment with onartuzumab plus erlotinib demonstrated improved PFS and OS, although the drug combination failed in a subsequent phase III trial [92]. A landmark study of 4,622 NSCLC patients with ALK rearrangement or MET amplification, of which 16 exhibited both alterations. The ORR was 86.7% in that subset of patients, suggesting a role of MET TKI treatment in MET-expressing tumors [93]. METROS, a phase II clinical trial, investigated the use of crizotinib—a multikinase inhibitor against ALK, ROS1, and MET—in MET or ROS1-positive pretreated, advanced NSCLC [94]. The trial confirmed anti-tumor activity with an ORR of 27%, a median PFS of 4.4 months, and an OS of 5.4 months. Another recent trial of 69 patients with MET exon 14 skipping mutated-NSCLC treated with crizotinib demonstrated encouraging data with an ORR of 32%, a median PFS of 7.3 months, and a median OS of 20.5 months [95]. Currently, there are several promising trials examining the therapeutic efficacy of other MET TKIs, such as cabozantinib (NCT03911193), cabozantinib (NCT02750215), REGN5093 (NCT04077099), tepotinib monotherapy (NCT02864992) plus osimertinib (NCT03940703), and telisotuzumab vedotin (NCT03539536). Of note, capmantinib was recently approved by the FDA for treatment of MET exon 14 skipping mutated-NSCLC due to results from the GEOMETRY mono-1 clinical trial [96].

### 4.5. KRAS

With an incidence rate of up to 22% in solid tumor malignancies, KRAS is one of the most prevalent oncogenic driver mutations in cancer [97]. In 1973, a murine sarcoma virus transformed mammalian genes into an oncogene in KRAS, and nearly a decade later, a human lung carcinoma cell line first showed human sequences analogous to the KRAS oncogenes in mice [98,99]. KRAS mutations in patients with advanced NSCLC are shown to have a poorer prognosis and often lack a response to standard therapies [100]. The KRAS oncoprotein triggers tumor cell growth and survival because it acts as a crucial mediator of intrasellar signaling pathways [101,102]. KRAS activating point mutations generally cause a loss of GTPase activity, and consequentially allow for the continuous activation of downstream signaling pathways such as MAPK and PI3K [103]. KRAS mutations have most recently been seen in exon 12 and 13 with the most common being G12C at 39%, G12V between 18–21%, and G12D between 17–18% [104]. In patients with lung adenocarcinoma, approximately 13% harbor a mutation of KRAS G12C [105]. KRAS mutant lung adenocarcinoma patients with concurrent inactivating mutations in the tumor suppressor genes of KEAP1 and STK11 have demonstrated lower clinical response rates treated with standard chemotherapy or immunotherapies [30,106]. KRAS G12C small molecule inhibitor, AMG-510, is the first in-human, phase I investigation in patients with advanced KRAS G12C solid tumors (NCT03600883). Taken orally, once daily, AMG-510 is a specific and irreversible inhibitor of KRAS G12C by fixing the guanosine diphosphate-bound state of the protein at an His95 groove in the P2 pocket of KRAS. Overall, the treatment was well tolerated. Twenty-two patients with results were presented and six of these patients had NSCLC. The median duration of treatment was 9.7 weeks. Two patients experienced a partial response and two had stable disease after six weeks [27,107]. Several other inhibitors including MRTX849 (NCT03785249) that target KRAS G12C and other KRAS mutation variants are currently being investigated in clinical trials.

### 4.6. Future Directions

The advantage of FFTs is that they are quick and accurate decisions based on limited information. This is useful in the world of oncology as unlike guidelines and pathways, FFTs do not restrict physicians to “on-pathway” and “off-pathway” decisions. FFTs incorporate physician’s “instinct” and “personal expertise” to arrive at a quick decision most beneficial to the patient. While this study was limited by sample size and a relatively focused cohort to one community oncology practice, the lesson of a standardized FFT frameworks have been shown to be applicable to not only stage IV lung cancer, but also to the early stage setting in the future. This study was limited to patients from one community practice, with a majority of patients demonstrating actionable mutations which does not completely represent the comprehensive population in community practice. Evaluating a larger cohort would be needed to further understand other treatment paradigms in NSCLC. However, in the future, it will be important to standardize the utilization of these FFTs to a usable application that physicians can refer to when reviewing their cases. This application would be most beneficial to community sites, as it would be a direct translation of the academic model to the community and would guide physicians towards the accurate decision without sacrificing their expertise. At the same time, we understand that the complexity of oncology practice varies from the cancer and the stage of the disease. Therefore, it will be important in certain situations to apply not only FFT heuristics, but also to incorporate multi-layered decisions of convolutional neural networks [108] and the quick capabilities of fast-and-frugal decision trees [33,35] into a hybrid model, conditional neural network. This would allow for the model to utilize the efficiency of fast-and-frugal decision trees and the accuracy of convolutional neural networks in a unified model.

## Figures and Tables

**Table 1:** Patient characteristics.

Patient Characteristics	N = 11
Median Age	
Year	70.5
Range	50–85
Sex	
Male	7
Female	4
Ethnicity	
White	6
Asian	4
Hispanic/Latino	1
Smoking Status	
Never smoker	6
Former smoker	5
Average pack year	10.2
Histology	
Adenocarcinoma	11
Symptoms	
Cough	5
Sputum production	1
Shortness of breath/dyspnea	3
Weight loss	3
Chest pain	1
Other	2
Performance status	
0	4
1	7
Clinical staging	
IIIA	1
IIIB	1
IVA	6
IVB	3
Molecular driver mutation status (positive)	
EGFR	5
KRAS	2
ALK	2
MET	1
RET	1
PD-L1 Status	
Positive ≥1%	5
Negative <1%	4
Not applicable	2
